# Commuting and communication: An investigation of taxi drivers’ experiences, attitudes and beliefs about passengers with communication disorders

**DOI:** 10.4102/ajod.v4i1.91

**Published:** 2015-02-06

**Authors:** Sianne Green, Munyane Mophosho, Katijah Khoza-Shangase

**Affiliations:** 1 Department of Speech Pathology and Audiology, University of the Witwatersrand, South Africa

## Abstract

**Background:**

One of the most popular means of public transport within South Africa is mini-bus taxis.

**Objectives:**

As South Africa is made up of diverse cultures, religions and beliefs, the aim of this study was to explore Johannesburg based taxi drivers’ experiences of beliefs about, and attitudes towards passengers who have a communication disability.

**Method:**

Semi-structured interviews were conducted with 10 mini-bus taxi drivers.

**Results:**

Interviews revealed that almost all the taxi drivers had encountered passengers with a communication disability, and had an awareness of passengers with a hearing disability as opposed to a speech disability. Furthermore mini-bus taxi drivers generally held a positive view of their passengers with a communication disability.

**Conclusion:**

Study findings contribute to existing literature within the fields of speech pathology and audiology, advocacy groups and policy makers, particularly research studies on participation experiences of persons with communication disabilities related to transportation access. The results of the study should also provide a foundation for disability policy development initiatives with the aim of increasing levels of public awareness.

## Introduction

People with communication disabilities face a variety of challenges in accessing everyday services, including the use of mini-bus taxis as a form of transport. The most popular mode of public transportation in South Africa is the mini-bus taxi: at least 65% of all public transport users utilise this service (Van Zyl 2009). These numbers indicate that over 14 million South Africans travel on mini-bus taxis daily, spending an average of 65 minutes commuting with an average of 2.3 taxi trips daily (Walters 2008). With these significant numbers in mind, one has no choice but to speculate and question how the 0.7% and 0.2% of the South African population with hearing impairments and communication disorders are affected in terms of access to taxi usage (Schneider & Couper 2007). A study by Mashiri *et al*. (2010) found taxis to be the primary form of public transportation for individuals with sensory impairments in South Africa.

The American Speech-Language-Hearing Association (1993) defines communication disorder as an impairment in an individual's capability to receive, send, process and, or, understand concepts, verbal and non-verbal, as well as visual symbol systems. Communication disorders involve hearing, speech and, or, language disorders. An individual with a communication disorder may present with a combination of these signs and symptoms, varying in severity. Speech disorders refer to disorders of articulation, fluency and voice whilst language disorders is an umbrella term for disorders of phonology, morphology, syntax, semantics and pragmatics. Hearing disorders, both deaf and hard of hearing statuses, are recognised as implicated in communication disorders.

The *World Report on Disability* (World Health Organization – [WHO] 2011) emphasises the significance of access to transport, especially for people with disabilities. Transport is the means by which people with disabilities have independent access to employment, necessary health care facilities, education and social and recreational events. Without accessible public transportation, people with disabilities will continue to be excluded from various services and social participation.

## The ICF and environmental factors

The World Health Organization's International Classification of Functioning, Disability, and Health (ICF) – 2001 – emphasises the central, influential roles environmental factors play in communication. The course of intervention of speech and language therapy should consider and address the influence these factors have on clients with communication disorders. Despite this, many speech-language pathologists and audiologists continue to neglect this aspect of therapy (Alant 2005). This scenario continues to change though, with a number of speech-language pathologists now including the impact as well as modifications of the environment to ensure efficacious care of their clients. It is for this reason that the researchers support the assertion that therapy cannot address environmental factors before adequate information is gathered regarding the various environmental factors that either support or impede client participation (Howe, Worrall & Hickson 2008). Because of the documented increased reliance on public transportation of people with certain communication disorders (Ashton *et al*. 2008), and the predominant use of mini-bus taxis in South Africa (Van Zyl 2009), this study set out to investigate taxi drivers’ experiences of, attitudes towards, and beliefs about passengers with communication disorders, in order to identify significant environmental factors influencing taxi usage within the South African context.

The ICF understands disability as a dynamic interaction between the individual's personal and environmental factors (WHO 2001). In this instance, personal factors refer to intrinsic features not pertaining to the health condition, whereas environmental factors are ‘all aspects of the external or extrinsic world that form the context of an individual's life and, as such, have an impact on that person's functioning’ (WHO 2001:213). Specifically, environmental factors involve the individual's physical surroundings, laws and legislatives, social and communicative encounters and society's attitudes and feelings towards the specific health condition (WHO 2001). Therefore, the environment plays a crucial role in relation to how individuals living with a communication disorder access resources in the community; and in this study, how they access public transportation.

### Environmental access and inclusion of persons with communication disability

In the ICF model, environmental factors exist in both negative (barriers) and positive (facilitators) forms. Howe *et al*. (2008) argue that there is little published evidence explaining the significant environmental factors that either support or impede the social participation of people with communication impairments. Whilst measurement tools that focus on environmental factors have been developed, very few have been developed in the area of communication disorders (Rimmer *et al*. 2004).

Watermeyer (2006) posits that many opportunities are unreachable because of the lack of safe and accessible transport and not as a result of the characteristics of an individuals’ disability. Previous studies have focused on the general population, on the elderly, and on people with visual and physical impairments (Jensen, Iwarsson & Staehl 2002). However, these studies disregard the barriers induced by communication disorders within the accessibility model of the ICF (Ashton *et al*. 2008). A study conducted by Mashiri *et al*. (2010) investigated provision of accessible public transportation in South Africa. One of the findings revealed that taxi drivers have difficulty communicating with ‘passengers with hearing impairments and those who cannot speak’ (Mashiri *et al*. 2010:7).

### Disability, access and transport in South Africa

The South African Human Rights Commission (SAHRC) provided a national mandate to both protect and secure the rights of all South African citizens, particularly those citizens vulnerable to compromised fundamental rights (Nhlapho, Watermeyer & Schneider 2006). Since the advent of democracy, South Africa has been active in promoting the rights of disabled people and is a signatory to the United Nations Convention on the Rights of Persons with Disabilities (UNCRPD). Despite policy commitments, delivery in terms of changes in the lives of disabled South Africans has, however, not always been as comprehensive as would have been wished (Nhlapo *et al*. 2006).

### Communication disability and transport access

Many individuals with a hearing impairment receive low incomes or rely on grants, creating a greater reliance on public transport (Lennie 2005). Research has described the communication between people with a hearing impairment and those without as difficult and problematic (Jaworski & Stephens 1998). In addition to the negative stigma associated with hearing loss, and the aggravation of repeatedly having to tell or remind people about their hearing impairment, many people with a hearing impairment commonly refuse to admit to their difficulties and subsequently avoid asking for assistance (Lennie 2005). Brennan and Bally (2007) explain that individuals with hearing loss commonly have difficulties communicating against loud background noise as well as with speech discrimination and anxiety associated with hearing loss. These factors may surface in the commonly high noise-level mini-bus taxi environment. Communication challenges may result from a variety of causes including dysarthria, stuttering, and problems communicating as a result of hearing impairments.

This study is important for advocacy for individuals with communication disorders and to find suitable methods for speech-language pathologists and audiologists to prepare their patients for using taxis.

## Methodology

### Research design

This study adopted a qualitative research design consisting of semi-structured interviews to attain the data which was focused on taxi drivers’ experiences with their passengers who have a communication disorder to extract in-depth information from a small number of participants (Webster & Mertova 2007). This design allowed the participants to describe their experiences, beliefs and attitudes (French, Reynolds & Swain 2001) as well as allowing the researchers to explore where and why knowledge and practice may be absent in this area (Marshall & Rossman 2011).

**TABLE 1:** The demographic profile of participants.

Participant	Age	Gender	Languages	Duration as a taxi driver (years)	Rank
1	32	M	isiZulu, Tswana, English	2	Illovo
2	30	M	Sepedi, isiZulu, English	5	Illovo
3	22	M	Sotho, Sepedi, isiXhosa, isiZulu, English	3	Waverley
4	28	M	isiZulu, Tswana, English	4	CMJAH
5	31	M	All official languages of South Africa	2	CMJAH
6	39	M	English, isiZulu, Ndebele and isiXhosa	3	Waverley
7	30	M	isiZulu, English, isiXhosa, Sepedi, Tswana	5	Illovo
8	37	M	isiZulu, isiXhosa, Sotho	4	CHBara
9	42	M	isiZulu, isiZulu, Sotho	15	CHBara
10	40	M	Xitsonga, isiZulu, Venda, Sepedi	5	CHBara

### Participants

The participants of this study consisted of 10 taxi drivers who worked at one of the following taxi ranks in Gauteng: Chris Hani Baragwanath Academic Hospital (CHBara), Illovo, Waverley and Charlotte Maxeke Johannesburg Academic Hospital (CMJAH). All had to have had experience with a passenger with a communication disorder.

All 11 South African official languages were represented amongst the participants – corresponding to the cultures, beliefs and attitudes associated with each language – although not every participant spoke all 11 languages. Additionally, the participants were chosen from four different taxi ranks in order to provide a larger representation of the population.

## Data collection, procedures and analysis

### Research instrument

Data were collected through an interview process. The interviews were semi-structured and consisted of both open-ended and closed-ended questions, allowing for both descriptive and specific information to be obtained (Kroll 2008). The location of each interview differed according to the participants’ preference, including inside their taxis and quiet areas within each taxi rank. Participants were given the choice of having the interview conducted in English or in their language of choice with the aid of an interpreter. Only one participant chose to have an isiXhosa interpreter present during the interview and the participant utilised both English and isiXhosa to express himself. The interpreter was trained by the researcher prior to the interviews, explaining ethical implications involved in research and data collection, the significance of translating verbatim, and non-bias on comments by the participants during the interview. The interpreter translated verbatim during the interview, allowing the researcher to probe.

The interview questions covered the following domains: participant information, awareness and knowledge of communication disorders, questions about their experiences with passengers with communication problems, and attitudes towards them.

### Pilot study

A pilot study to pre-test the research tool and method was conducted with a sub-sample of two participants. It was fundamental in assuring content-validity – that all questions in the interview were appropriate to extract desired data – and ensuring that interview questions were understood by the participants (Breakwell, Hammond & Five-Schaw 2000). The pilot study led to the replacement of the terms ‘communication disorders’ and ‘language problems’ with ‘speech problems’ as a trend was that language problems referred to language barriers.

### Procedure

Ethics clearance was obtained from Wits University Human Research Ethics Committee. Participant codes were used on all data collection forms to ensure confidentiality, with data accessed by the researchers only.

The first author spent a few hours in a taxi to familiarise herself with the way in which taxis operate. The taxi drivers were initially unwilling, but agreed once they understood the aims of the research. The type of taxi driver that was interviewed was seen by the researcher to be more patient than others who would not wait in the rank to be interviewed. The findings of this study should be interpreted with the knowledge that participants may also be more patient with passengers who have a communication disability.

### Data analysis

The researcher audio recorded each interview then orthographically transcribed the data to achieve optimal documentation of the information given (Poland 2001). At least 25% of the transcriptions were re-transcribed by an independent observer to ensure accuracy and integrity.

Thematic content analysis was implemented for analysis (Patton 2002). The coding scheme employed the use of categorisation to group focal themes in the findings. Following thematic content analysis, the researcher appropriately linked the taxi drivers’ experiences to structural organisation (Brbich 2007), and further compared and contrasted the data with available literature in the field. An independent rater (an experienced speech-language pathologist) checked the ratings made by the researcher.

## Results and discussion

### Taxi drivers’ awareness and knowledge of speech and language disorders

Only two participants claimed to know what a speech disorder was. One participant said that he had not had a passenger with a speech problem on his taxi but later described an experience in which the passenger being described had a speech problem. This example typified the lack of awareness amongst these participants. It was found that when asked to explain what a speech problem is, only four of the participants provided an explanation. Although accurate, these descriptions lacked detail about speech disorders: ‘[*I*]t is a situation whereby someone can't actually speak …’; ‘… if you don't know how to pronounce things or like you don't even know how to talk’; ‘… somebody that can't speak totally’.

Only one participant acknowledged that although persons with a speech disorder may have difficulty articulating their message, or cannot produce speech, these individuals do not essentially have an impairment in understanding. This participant described an individual with a hearing impairment as an individual who, ‘… can understand me when I am talking but he cannot pronounce words like that’, and stated that he ‘… had someone on this taxi who didn't know how to talk but he was able to hear’.

Another trend was that these passengers were said to use sign language. This demonstrated an understanding that people with hearing impairments are individuals who ‘… can't actually speak and maybe at the end of the day they use sign language or something’. It seemed that although the participants accurately described a speech disorder as an inability or difficulty with speaking, the participants associated speech disorders with hearing impairment in the main.

### Taxi drivers’ awareness and knowledge of hearing impairments

Hearing impairments were described as varied, ranging from hard of hearing to deaf: ‘At times there are some people who can't hear at all and there are some people who like can hear but the sound is very far away’.

Furthermore the participants’ awareness of the communication effects of a hearing impairment was evident. One participant mentioned that, ‘… you need to speak louder if they have that problem and some you need to use sign language’.

### Taxi drivers’ attitudes and beliefs

One participant mentioned that individuals, ‘… who can't hear it's painful because sometimes you speak to someone that doesn't hear and maybe you don't know that person can't hear’. The same participant explained:

‘[*I*]f somebody can't hear you so it's a problem because you can't communicate with that person because he can't hear you … if the person doesn't know how to speak, he can't communicate’.

The belief of this participant seemed to be that the only mode of communication is verbal communication. Although this participant has described individuals with a hearing impairment as having a ‘painful’ experience he does not have any negative beliefs about the cause of these impairments and believes them to be ‘just medical’ in nature.

All other participants demonstrated positive attitudes toward people with communication disorders. Individuals with communication disorders were not seen to be cursed or bad as this was ‘… just how they were born’. The majority of participants reinforced that these individuals had not carried out anything negative that resulted in a communication disorder, as ‘… they never made themselves like that’. The conclusion was that:

‘… the cause of that we don't know because there are certain thing that come in life that might make somebody to come out blind, come out without even hearing anything, can't even talk or open their mouth’.

Despite the cause being unknown, individuals with a communication disorder were described as ‘normal people’. The underlying belief seen was that ‘… they are people and we mustn't undermine them’:

‘You see if you are a human being, you know there are many people that are different, there are different characters. So the other people they don't speak, some they speak, some they speak another language you don't know. Some they just speaking signs like I'm telling you’.

‘How could they be bad, how could they be bad people. It's not like, yah, if they did that to themselves I would say, yah well, they are bad people, but it happened you check. It's something that just, that just happened really so would you blame nature for what’?

### Experiences of passengers with a communication disorder

There were no reported occurrences whereby an individual with a communication disorder caused an inconvenience on a taxi. All participants were in agreement about these passengers: ‘[*I*]t's not a problem’. The other passengers on these journeys worked together in assisting the individuals with communication disorders where necessary. One participant added, ‘… that's what you do, make sure that our passengers work together till they get to their destinations’.

When one participant was asked how the other passengers on his taxi responded to the passenger with the communication disorder, he replied: ‘I never realised how all the passengers reacted’. Other passengers did not draw attention to the individual with a communication disorder on this particular taxi and it probably resulted in the problem-free journey.

### Techniques used to communicate desired destination

The most frequently used, and most highly recommended technique, was writing down the desired destination for the driver to read. One participant mentioned that a passenger on his taxi was able to communicate his destination by tapping another passenger on the shoulder who told the driver to stop the taxi. Another participant explained that a passenger on his taxi was able to communicate his destination by making a ‘noise’, at which the driver knew to stop the taxi for him. Another technique was speaking to the taxi marshal for assistance in using the appropriate taxi for their destination. Although recommended by one of the participants, this is a technique only available to passengers at taxi ranks. One participant suggested over-articulating the destination – ‘… try and spell … on his mouth’ – as a technique for individuals who do ‘… not know how to write’.

## Discussion of themes in the data

### Feelings of responsibility to passengers with a communication disorder

Participants felt a sense of responsibility to their passengers with communication disorders and other disabilities. One participant illustrated this by stating:

‘If you are a driver, you need to understand your passengers, you need to provide them with a good service so it's a must for us taxi drivers, if someone come to us we need to understand that if he or she has a problem with hearing; there is something we must do for them’.

The participants explained that people with communication disorders and varying types of disabilities are welcomed on their taxis and are further encouraged to continue using this form of transport. The participants described that, as taxi drivers, they are responsible for ensuring that all their passengers, those with and those without disabilities, receive the best service. ‘We will keep on providing our best especially on the people who are being disabled someway’.

The document *Transport for Disabled People* outlines this behaviour as it stipulates that all individuals, despite the type or severity of their disability, have the right to accessible public transport (National Department of Transport [NDOT] 2000). The participants said that they needed to take the necessary measures in ensuring individuals with a communication disorder are understood on the taxi, that it is their responsibility to combat the communication barriers and find alternative modes of communication that are beneficial to both themselves and passengers with communication disorders.

The participants in this study commented on their role of working in an industry serving the public. A common theme was significance patience and trying different techniques in supporting individuals with communication disorders. One participant added that as taxi drivers, ‘… [*We*] really make sure that we help where we can like get things right’. The ‘patience’ theme correlates with literature that explains impatience as a critical barrier to successful communication (Howe *et al*. 2008). Furthermore, the removal of time pressure improves communication for individuals with certain communication disorders (Dickson *et al*. 2008; Manning 2000). ‘At the end of the day we assist such a person’.

### The notion that individuals with communication disorders are ‘normal’ people

Downs (2011) explained that the effect of the individuals’ communication disorder is not confined by the physical manifestation of the disorder, as the emotional effects may be far more severe. This includes low self-esteem, feelings of worthlessness, and depression (Kent 2004). These harmful emotions have a directly negative impact on the individuals’ quality of life.

Individuals with communication disabilities have described the negative stigma attached to their disability that has subsequently resulted in these individuals avoiding events or circumstances that require communication with other individuals, such as hearing-impaired people avoiding interaction with hearing individuals (Lennie 2005). The participants in this study consistently described individuals with communication disorders as ‘normal people’, with one adding: ‘… [*T*]hey are people you know’. These remarks had no attachments of a negative stigma to people with communication disabilities.

Another common emotional attribute experienced by individuals with communication disorders is feeling socially isolated (Tanner 2008), feeling alienated and different to the rest of society (Dickson *et al*. 2008). These beliefs and feelings affect the individual's level of participation within society (Siperstein *et al*. 2006). A fundamental theme in each interview was that individuals with communication disorders, ‘… are same like us … We are the same people’. This view contradicts the views held by individuals with the communication disorders as highlighted by Tanner (2008).

Disability is context created, as societal attitudes as well as the infrastructure in place, or the lack thereof, contribute to the experience of being disabled (Swartz & Schneider 2006). With the approach of ‘… they are people and they are same like us’ and ’… they are people who must be treated like other people’, the participants in this study articulated that individuals with communication disorders should not feel isolated or separate from taxi drivers and the other passengers. Furthermore the South African Constitution and the Integrated National Disability Strategy (INDS 1997) state that all people are equal regardless of race, gender or disability. This theme of ‘sameness’ correlates and supports democratic principles of equality: ‘… [*B*]ecause they are human beings like us. We are the same. They are normal beings like us’.

### Level of independence or taxi usage

The goal of ICF is to provide a standardised framework in describing human functioning and disability as an important component of health (Ross & Deverell 2004). The ICF defines an individual's health in terms of anatomical and physiological features, as an individual's life is influenced by environmental and personal factors as well. The classification involves any disturbances in terms of ‘functional states’ associated with health conditions at bodily, individual and societal levels (Ross & Deverell 2004:14). Regarding participation and independence, three main themes were revealed in this study: encouragement to use taxis; the ability for individuals with communication disorders to independently pay the taxi fare; and the debate on whether or not an individual with a communication disorder could use taxis independently.

Despite previous research that has found people with disabilities to be a challenge for taxi drivers, the participants in this study encouraged the continuous use of taxis as a means of public transport: ‘They must continue getting to our taxis just like that’. One participant added:

‘I think sign language is the right thing to do because you can't go with your child or your friend everyday so that they can do anything on taxi. Sometimes you have other things to do. So he have to go alone so it's better to use the sign languages’.

This statement also expressed the participant's belief that individuals with a communication disorder should use taxis as their means of access to functional participation.

Being able to independently pay the taxi fare impacts on an individual's level of independence. The participants commonly reported that these individuals ‘can count the money’, illustrating that individuals with a communication disorder do not need to rely on assistance in this activity. One participant added that he uses gestures to tell individuals with a communication disorder how much the taxi fare is.

There were some differing viewpoints on whether individuals with communication disorders should use taxis independently or if they should be accompanied. Those participants in favour of independence explained that, as the taxi driver, they will assist these individuals, whilst others added that with appropriate measures, such as the knowledge and use of sign language, these individuals are able to independently use public transport. On the other hand, some participants expressed the view that individuals with a communication disorder need assistance: ‘I haven't had one on his own. It is rare, one in ten, it is very rare’. Furthermore, some of the participants in this study ‘… urge those people not to go by themselves’. The belief that individuals with communication disorders should be accompanied on taxis is contradictory to the principles of full participation and independence in society for all, as outlined in the Constitution.

## Conclusion

This study set out to determine taxi drivers’ experiences with passengers with communication disorders. Findings revealed greater knowledge and awareness of hearing impairments compared to speech impairments; an interesting finding since hearing impairment is dubbed the ‘silent and invisible’ disability. However, in the context of communication disorders, it can be argued that hearing impairment can be the more ‘visible’ disability as individuals will utilise devices such as hearing aids and, or, use sign language to communicate. It is evident that this understanding of communication disorder as comprising mainly the use of sign language, with no distinction between a speech and a communication disorder, influenced their experiences with people who have communication challenges. Participants’ understanding of sign language was that it is used equally by both individuals with speech and, or, hearing impairments. Whilst some individuals with speech impairments utilise sign language, the participants assumed that individuals who use sign language have a communication disability. This perception detracts from the fact that sign language has been afforded equal status in relation to the other languages spoken in the country – and is not only used by people with communication disorders. This finding therefore has implications for advocacy groups (for example the Pan South African Language Board, and Deaf Federation of South Africa) concerned with establishing and promoting use of all languages in the country. Moreover, this finding highlights an implication for clinical intervention which is the openness to sign language as a mode of communication by this population.

The fact that participants viewed individuals with communication disorders as equals, with no negative stigma to a communication disorder, was a positive finding. Participants regarded individuals with communication disorders as ‘good’, normal people. This finding, arguably, indicates a positive and embracing culture rather than a negative and discriminatory one, facilitating participation and inclusion.

Differing viewpoints on beliefs regarding individuals with communication disorders’ independent use of taxis were found. Despite some participants’ strong stance of being accompanied whilst using taxis, none of the participants believed that individuals with communication disorders should not have equal access and usage of taxis. In accordance with various laws and legislation in South Africa, many participants acknowledged their responsibility to their passengers with communication disorders and other disabilities. This finding was thought to possibly be influenced by the milder degrees of communication impairments that the participants might have been using as their points of reference: possibly, views might have been different had they dealt with individuals with severe communication disorders.

Regardless of the severity of the communication disorder, the most recommended technique to use when communicating one's desired destination to a taxi driver in the current study was writing the destination on paper. Although this is a beneficial proactive technique for both taxi drivers and individuals with varying communication disorders, it does limit the participation level of the individual to only indicating destination. It also assumes that the written language skills and motoric skills required for writing are intact; this is if literacy is not an issue. A communication system that also allows for use of pictures is crucial in such a context; this is now readily available to a majority of people with access to cell-phones with mobile applications. Clinical interventions with individuals with communication disorders should incorporate such techniques to increase participation in this context.

Current findings should be interpreted within the identified limitations in the design of the study. The small sample size, as well as restriction to the Gauteng province, limits generalisation of the findings across the South African population. It is also important to highlight that participants in the study were seen as more patient than the other taxi operators.

Nonetheless, the results of this study provide baseline knowledge of taxi drivers’ experiences, attitudes and knowledge of individuals with communication disorders, with implications for future research. The findings of this study also provide speech-language pathologists and audiologists with useful information regarding areas in which advocacy is required. This includes creating an awareness of different speech impairments and ensuring the execution of the INDS's and South African Constitutions’ documented rights and transport legislations for individuals with disabilities.

Internationally, public transportation is moving forward in identifying individuals with disabilities as important: Crewe and Zola (2001) explained that developments are in the process of accommodating individuals with disabilities for both long and short distance travelling. However, recommendations have been made largely to accommodate physically disabled individuals. When considering communication disorders, public awareness should be promoted so that advances can be made in accommodating individuals with communication disorders.
